# Siftings Saved

**Published:** 1893-11

**Authors:** I. Dever

**Affiliations:** Clinton, N. Y.


### SIFTINGS SAVED.

I. Dever, M. D., Clinton, N. Y.

Much has been said and written in the last twenty years about the necessity of sifting the homœopathic materia medica—the supposed necessary operation having for its object the separation of what those would-be sifters regard as important or characteristic symptoms from those of but little or no importance.

A second sober thought is hardly necessary to impress the earnest, careful student of materia medica with the herculean nature of such an undertaking, proposed by a class of superficial-thinking men, whose opinions fall far below the dignity of just criticism.

We have often heard them say "the homœopathic materia medica is too complicated, and should be simplified by expunging the less-important symptoms." They would lay the axe at the very root of the science, and destroy that which they fail to comprehend; for what homœopathic prescriber does not know that the expunging of one single symptom from the written record of a remedy would invalidate the truth of the record and destroy the value of the symptom, which can only be thoroughly known by the physician after he has verified it by prescribing it and with it curing the sick. I have always been curious to know what particular class of symptoms our would-be pruners of the homœopathic materia medica would be willing to lop off as worthless boughs or winnow out as useless chaff.

How this purification is to be brought about is a question worthy our consideration, as they would certainly not attempt a reproving of all of our well-proven remedies. Such provings, if instituted and carried out in good faith, would, under the same circumstances, be a verification of the truth of the *Materia Medica Pura*, for they would repeat themselves as often as such provings should be instituted in the spirit of truth. Now I have it! I think they had better appoint a committee who shall tell us just what symptoms are to be regarded as chaff and which as wheat, and that will settle the question, just as the question of potency has been settled in the minds of those who cannot find any results from a potency higher than the twelfth. But, as there are none so blind as those who will not see, we will still have to be numbered with those who cannot see it in that light; consequently, we are left to grovel after siftings with which to cure our patients. Now, as we have a hankering after siftings, we will present two or three cases cured by symptoms which would never have found a place in the homœopathic materia medica were the provings made with less care and patient painstaking following of the directions to be found in relation to proving the medicines on the healthy. (See Hahnemann's *Organon.*)

The first case which I will here present was that of a man fifty years old, who had many bad habits not supposed to have been taught him in Sunday-school. On his fiftieth birthday, which was in February, he went to Utica, and, as bad company is much more readily found than good, he fell in with a lot of drinking men and got gloriously drunk. If there is such a thing as a glorious drunk, he had it, for he drank and exposed himself until he fell sick with a very bad sore throat; he attended himself with liniments and gargles until he became thoroughly frightened; then he sent for me.

I found his throat terribly swollen; pain on deglutition; profuse discharge of fetid saliva. I could not examine the throat internally on account of the swelling; but where I could see the mucous membrane it was slick and glistened. When he opened his mouth the saliva would adhere to his mouth, and the little threads would extend from the tongue to the teeth and soft palate. The tongue was coated a dirty white. I prescribed Merc. Jod. Rub., some pellets in water, a dose to be taken every hour for three hours, and no medicine to follow. The next day he was better and went out and down-town. Two days after his trip down-town he sent for me, and I found the last state of the man worse than the first. He told me that the pain in the throat *began in the right side, went to the left, and then returned to the right.* The pain was greatly aggravated by empty swallowing. I gave him one dose of Lac-can.$^{1m}$, dry, on his tongue; no more medicine, and after that day no more sore throat.

A short time since I was called to treat a lady with a sore throat. She gave me the following history of the case: She stated that she had had attacks of quinsy, which always came with her monthly periods, and that her doctor, an allopathic, always treated her with gargles and Quinine; she recovered from time to time, but had about made up her mind that she would get along as well without him, and had concluded to try my treatment. I prescribed Mag-carb.$^{1m}$, one dose, with a second powder to be taken providing she felt symptoms of a return; the next time she had her monthly sickness she felt symptoms and took the powder, but has had no more quinsy and is greatly better in every way.

I might continue this paper, but as I have only presented it in the interest of the siftings which we will do well to save, I will close.

